# Telomere chromatin establishment and its maintenance during mammalian development

**DOI:** 10.1007/s00412-017-0656-3

**Published:** 2017-12-18

**Authors:** Mathieu Tardat, Jérôme Déjardin

**Affiliations:** Institute of Human Genetics, CNRS UMR 9002, 141 rue de la Cardonille, 34396 Montpellier, France

**Keywords:** Telomere, Chromatin, Development, Genome stability

## Abstract

Telomeres are specialized structures that evolved to protect the end of linear chromosomes from the action of the cell DNA damage machinery. They are composed of tandem arrays of repeated DNA sequences with a specific heterochromatic organization. The length of telomeric repeats is dynamically regulated and can be affected by changes in the telomere chromatin structure. When telomeres are not properly controlled, the resulting chromosomal alterations can induce genomic instability and ultimately the development of human diseases, such as cancer. Therefore, proper establishment, regulation, and maintenance of the telomere chromatin structure are required for cell homeostasis. Here, we review the current knowledge on telomeric chromatin dynamics during cell division and early development in mammals, and how its proper regulation safeguards genome stability.

## Introduction

**Fig. 1:**
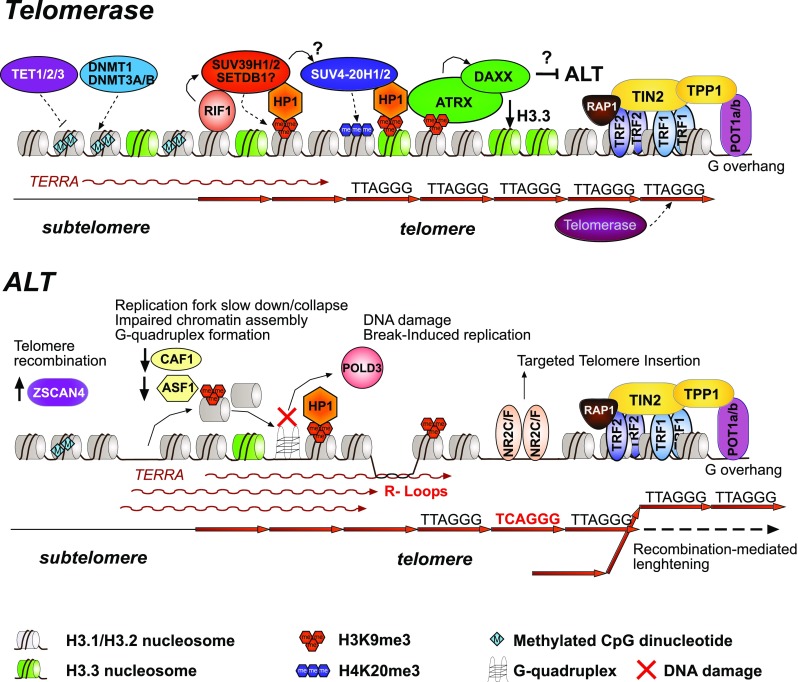
Chromatin structure of human and mouse telomeres. Telomeric chromatin is organized in a closed state with repressive histone posttranslational modifications (PTM), similar to those observed at pericentric heterochromatin (PCH) domains. Telomeric nucleosomes are labeled with H3K9me3 and possibly H4K20me3, while subtelomeric DNA is also methylated at CpG dinucleotides. H3.3-containing nucleosomes are provided by the ATRX/DAXX complex. Although telomeric chromatin is heterochromatic, subtelomeric regions contain promoters that promote the transcription of the long non-coding RNA TERRA. Telomere length is maintained by the action of the enzyme telomerase that catalyzes the addition of extra TTAGGG repeats to chromosome ends. Shelterin complexes bind along the telomere and protect from recognition by the DNA damage pathway. For graphical clarity, only one shelterin complex (TRF1, TRF2, POT1a/b, TPP1, and TIN2) is shown (top). In a subset of cancer cells, telomerase is not used to elongate telomeres. Instead, alternative lengthening of telomere (or ALT), allows recombination-mediated elongation. *ATRX* is frequently lost in ALT cells. Repressive marks are reduced, and TERRA RNA is more abundant, leading to putative recombinogenic DNA-RNA hybrids (R-loops). Variant repeats in ALT cells can induce binding of NR2C/F transcription factors to telomeres, leading to chromosomal rearrangements and genomic instability (bottom)

In eukaryotes, the genome is organized in different compaction states. Schematically, euchromatin, which defines the open compaction state, contains active genes, whereas heterochromatin, which is the repressive compacted chromatin, includes gene-poor regions and different types of repeated elements with a specific chromatin structure (Nishibuchi and Déjardin 2017). The ends of linear chromosomes, or telomeres, are protected from recognition by the DNA damage response (DDR) machinery. This prevents chromosome end-to-end fusion and cell death (Palm and de Lange 2008). In mammals, telomeres are wrapped with nucleosomes and form a heterochromatin structure (Schoeftner and Blasco 2010). Telomeres consist of an array of tandem repetitions of the hexanucleotide TTAGGG motif that spans over several kilobases (10-15 kb in humans and up to 50–100 kb in laboratory mice). Because of the semi-conservative process of DNA replication, telomeric DNA shortens at each cell cycle, a phenomenon known as the “end replication problem” (i.e., the inability of DNA polymerases to fully replicate linear genomes) (Gilson and Géli 2007). Once telomeric DNA shortening reaches a critical point, this triggers the permanent exit from cell cycle, preventing the deleterious loss of genetic material encoded by the chromosome ends. However, telomeric DNA can be elongated by the telomerase enzyme or by alternative mechanisms (Cesare and Reddel 2010) (Fig. 1, upper panel). Here, we review the current knowledge on the role of canonical chromatin factors on telomere dynamics, particularly how the telomere chromatin structure regulates telomere maintenance in meiosis and in early development.

### Maintenance of telomere chromatin structure in mammalian cells

#### Components of telomeres that protect against DDR

The Shelterin complex is one of the main components of mammalian telomeres (Palm and de Lange 2008) and consists of six main proteins in mammals: TRF1, TRF2, POT1 (POT1a and POT1b in mice), TPP1, and TIN2 (Fig. 1). TRF1, TRF2, and POT1 bind to telomeric double- and single-stranded DNA, respectively. The current model of Shelterin assembly suggests that each complex binds independently to telomeric DNA repeats according to a “beads on a string” pattern, the length of which is proportional to the telomere length (Erdel et al. 2017). Then, Shelterin protects the telomere end from recognition by the DDR machinery, thus preventing deleterious end-to-end chromosomal fusions (van Steensel et al. 1998). TRF2 is a key player in telomeric DNA protection by regulating T-loop formation at telomeres and by suppressing DDR (reviewed in Feuerhahn et al. 2015). This last function could depend on Shelterin-mediated chromatin compaction, thereby preventing telomere expansion and hindering its recognition by the DDR machinery (Bandaria et al. 2016). However, two recent studies using stochastic optical reconstruction microscopy (STORM) and assay for transposase-accessible chromatin with high-throughput sequencing (ATAC-seq) showed robust DDR localization at telomeres following co-depletion of TRF1 and TRF2, despite the fact that their depletion did not significantly affect telomeric chromatin compaction and accessibility (Timashev et al. 2017; Vancevska et al. 2017). This suggests that telomere recognition by DDR is most likely due to changes in telomeric chromatin structure and composition rather than to decompaction.

Moreover, in mammalian cells, telomeres and subtelomeric regions harbor specific histone posttranslational modifications (PTMs) and proteins that are typically found at pericentric heterochromatin (PCH), such as trimethylation of lysine 9 of histone H3 (H3K9me3), trimethylation of lysine 20 of histone H4 (H4K20me3), hypoacetylation of histone H3 and H4, and HP1 proteins (Schoeftner and Blasco 2010). However, H4K20me3 was not found at telomeres using mass spectrometry approaches (Saksouk et al. 2014). Subtelomeric regions that contain CpG dinucleotides are also methylated. Like PCH, telomeric DNA is considered to be in a closed and repressed heterochromatic environment. Nevertheless, transcription by RNA polymerase II is observed at subtelomeric regions, leading to the production of the long non-coding RNA TERRA that coats telomere ends (López de Silanes et al. 2014). Although its precise function remains enigmatic, TERRA was proposed to play several roles in telomere maintenance (reviewed in Azzalin and Lingner 2015).

#### ATRX and alternative lengthening of telomeres

By perturbing the chromatin state of telomeres, several studies demonstrated the critical influence of the chromatin structure on telomere homeostasis and length (O’Sullivan and Almouzni 2014). Stem cells and most cancer cells use telomerase to add de novo TTAGGG repeats and prevent telomere shortening in order to maintain high cell proliferation (Fig. 1, upper panel). However, a subset of cancer cells (about 10–15%) do not rely on telomerase activity but use a recombination-mediated alternative lengthening of telomere (ALT) mechanism (ALT cells) (Dunham et al. 2000) (Fig. 1, bottom panel). ALT cells can be identified by the presence of PML bodies that contain telomeric DNA, Shelterin proteins, DNA repair factors and chromatin proteins, and that are known as ALT-associated PML bodies (APBs). Other ALT features include telomere clustering, extrachromosomal DNA of telomeric repeats (both linear and circular DNA), telomere sister-chromatid exchange (T-SCE), telomere length heterogeneity, and absence of telomerase (O’Sullivan and Almouzni 2014). Although these features are often considered as ALT hallmarks, they may be observed also in non-ALT cells in some conditions. For instance, human embryonic stem (ES) cells contain extra-circular telomeric DNA that results from trimming of overly long telomeres (Rivera et al. 2016). Therefore, these characteristics are most likely to be the consequence rather than the causality of recombination occurring at telomeres. However, recombination is a requirement for ALT maintenance, because depletion of homologous recombination proteins impairs ALT and results in telomere shortening (Zhong et al. 2007). ALT cells contain variant telomeric repeats that are recognized by the nuclear orphan receptor NR2C/F that can promote chromosomal rearrangements (Déjardin and Kingston 2009; Marzec et al. 2015). Indeed, ALT-mediated telomere maintenance is associated with increased genomic instability.

Because of its occurrence in cancer cells, it is important to understand the molecular mechanisms and factors involved in ALT induction. Interestingly, absence of ATRX expression or localization at telomeres is a frequent feature of ALT cancer cell lines (Heaphy et al. 2011; Lovejoy et al. 2012) (Fig. 1). ATRX is a chromatin remodeler that contains a SWI/SNF2-type ATPase/helicase motif and a plant homeodomain-like zinc finger (Watson et al. 2015). ATRX forms a complex with the histone H3.3 chaperone DAXX that regulates histone loading at telomeres (Wong et al. 2010; Lewis et al. 2010). Strikingly, when ectopic ATRX is expressed in *ATRX*-null human osteosarcoma U2OS cells, the ALT pathway is reduced. This effect requires DAXX function (Clynes et al. 2015; Napier et al. 2015). Several hypotheses were proposed to explain how *ATRX* mutations promote ALT (reviewed in Amorim et al. 2016). ATRX is involved in the formation of repressed heterochromatin structures at several genomic loci, such as intracisternal A particle (IAP) retrotransposons and imprinted loci (Voon et al. 2015; Sadic et al. 2015). Loss of *ATRX* function leads to reduced incorporation of histone H3.3; therefore, chromatin marks cannot be maintained, presumably leading to telomere derepression and increased TERRA transcription (Udugama et al. 2015; Nguyen et al. 2017). Transcribed telomeric repeats form RNA-DNA hybrids (R-loops) that are supposed to promote the formation and stabilization of G-quadruplexes at telomeric repeats. As G-quadruplexes can impair replication fork progression, the increased rate of replication fork stalling might lead to DNA damage and drive homology search and recombination at telomeric regions (Arora et al. 2014).Another study suggested that in the absence of *ATRX*, the histone variant macroH2A1.1 binds to the poly(ADP-ribose) polymerase tankyrase 1, thus preventing its binding to telomeres and the resolution of cohesion between sister chromatids. Persistent cohesion would then promote recombination by T-SCE (Ramamoorthy and Smith 2015). However, activation of the ALT pathway might not depend only on ATRX. Indeed, *ATRX* inactivation in primary or immortalized cell lines does not trigger ALT (Napier et al. 2015). Moreover, ALT is more frequently observed in cancer cells of mesenchymal origin (Jiao et al. 2011; Heaphy et al. 2011). These observations suggest that additional chromatin or cellular events (e.g., DNA damage at telomeres) could be required to promote ALT in the absence of ATRX (Hu et al. 2016). For instance, co-depletion of the two paralogs of anti-silencing function 1 (*ASF1a* and *ASF1b*) leads to ALT (O’Sullivan et al. 2014). ASF1 is a histone chaperone of both H3.1/H3.3-H4 dimers that plays a critical role in nucleosome transfer during DNA replication. Thus, destabilization of nucleosomes at telomeres could affect the chromatin state, leading to ALT induction.

#### Safeguarding telomeric chromatin during DNA replication

DNA replication involves the faithful duplication of the genetic material and proper re-establishment of the parental chromatin states in both daughter strands. Chromatin reassembly after the replication fork passage requires efficient maintenance mechanisms to preserve the epigenetic information and cell identity. For instance, replication fork stalling might affect proper transfer of nucleosomes and maintenance of chromatin marks, thus impairing the binding of readers to their specific histone PTM (Groth et al. 2007; Jasencakova et al. 2010). Purification and analysis of the proteins present on chromatin associated with newly synthesized DNA showed that heterochromatic proteins, such as SUV39H1/2, DNMT1, and HP1, are readily found on nascent chromatin, while the re-establishment of H3K9me3 on parental and new histones occurs over several cell cycles (Alabert et al. 2014, 2015).

As telomeres resemble fragile sites, they may challenge the DNA replication machinery. Moreover, ALT cells undergo replication stress due to the presence of long, recombinogenic telomeres. Therefore, specific proteins are required for proper replication of the chromosome ends and for preserving genomic integrity (Higa et al. 2017). ATRX is critical for resolving DNA structures, such as G4-quadruplexes, that could induce replication fork stalling at repetitive regions of the genome, including telomeres (Law et al. 2010). As ATRX interacts with H3K9me3, through its ADD (ATRX-DNMT3-DNMT3L) domain, and with HP1, through a conserved motif (Iwase et al. 2011), improper maintenance of nucleosomes and histone PTM at telomeres might prevent ATRX binding, thus compromising telomeric DNA stability. Replication defects could impair ATRX loading and affect telomere maintenance. Several Shelterin components also participate in the proper maintenance of telomere stability by recruiting specialized enzymes to resolve deleterious secondary DNA structures that might form at telomeres. For instance, TRF1 and TRF2 associate with the helicases Werner syndrome RecQ helicase (WRN) and Bloom syndrome protein (BLM) that are involved in the resolution of G-quadruplexes and Holliday junctions. The functions of these two helicases do not fully overlap. WRN can promote the restart of collapsed forks by break-induced replication (BIR). WRN associates with SWI/SNF-related, matrix-associated, actin-dependent regulator of chromatin, subfamily A-like 1 (SMARCAL1), another helicase that interacts directly with replication protein A (RPA) and is involved in replication fork restart and genome stability (Ciccia et al. 2009). In the absence of *SMARCAL1*, collapsed replication forks at telomeres are converted to DNA double-strand breaks that promote end-to-end chromosome fusion and compromise cell viability (Cox et al. 2016). Thus, SMARCAL1, BLM, and WRN are essential factors to prevent replicative stress of both normal and ALT telomeric DNA. T-loops also represent a barrier to replication forks and must be unwound during DNA synthesis. This could be partly mediated by the helicase RTEL1 (Vannier et al. 2012; Sarek et al. 2015) because *RTEL1* loss induces replication fork stalling and C-circle accumulation.

Replication of chromosome ends is not only a matter of replicative stress management but also of preservation of the chromatin state. Indeed, subtelomeric regions contain genes and TERRA promoters, the activity of which must be regulated. Modifications in the chromatin status or DNA methylation levels of telomere and subtelomeres can alter TERRA expression (Schoeftner and Blasco 2008). Uncontrolled TERRA expression might favor the accumulation of RNA-DNA hybrid structures that could impair fork progression and stability. In mouse ES cells, the telomeric protein RIF1 associates with multiple H3K9 histone methyltransferases to maintain subtelomeric heterochromatin silencing (Dan et al. 2014). Upon *Rif1* knockdown, this heterochromatic structure is lost and the subtelomeric *Zscan4* gene is derepressed, leading to an overrecombination phenotype (*Zscan4* function is discussed below). Similarly, the epigenetic regulator structural maintenance of chromosomes (SMC) flexible hinge domain-containing 1 (SMCHD1) is involved in the silencing of subtelomeric *DUX4* repeats (Lemmers et al. 2012). In human cells, *DUX4* is localized in the subtelomeric region of chromosome 4q. This locus is composed of tandem repeats of 10 to 100 D4Z4 units organized in a condensed heterochromatic state in somatic cells. Genetic or chromatin alterations of the repeat array are associated with facioscapulohumeral dystrophy (FSHD) (reviewed in Daxinger et al. 2015). SMCHD1 is a non-canonical SMC protein identified in a screen for genes that induce a variegated expression pattern of a multi-copy transgene in the mouse (Blewitt et al. 2005). In *SMCHD1* heterozygous individuals, SMCHD1 levels are reduced and this was correlated with increased *DUX4* expression. Although many facets of SMCHD1 regulation are still under investigation, it appears essential for the maintenance of the heterochromatic state of tandem repeats, including telomeric DNA.

Thus, maintenance of the chromatin state of telomeres in daughter cells is vital for genome stability and cell identity. The interplay between chromatin regulators and the replication machinery or Shelterin proteins is an efficient system to perpetuate chromatin states as the cells divide. In the next chapters, we will discuss how chromosome ends are regulated during the drastic chromatin remodeling that occurs during germ cell differentiation and early embryonic development.

### Chromatin dynamics at telomeres during early mammalian development

#### Telomere structure in the germline

The germline has the critical task of producing the male and female gametes (i.e., spermatozoa and oocytes) through a process called gametogenesis. Importantly, during this process, male and female germ cells undergo meiosis, a specialized cell division characterized by one round of DNA synthesis followed by two rounds of cell division, resulting in the production of haploid gametes. During and after meiosis, germ cells undergo chromatin remodeling, leading to specific DNA and histone methylation patterns, and to histone to protamine exchange in male germ cells (Kota and Feil 2010). Perturbation of these processes can result in the reduction or loss of germ cells, leading to infertility, or in aneuploidy, leading to abortive pregnancies or congenital abnormalities in the newborn. Besides their role in protecting the chromosome ends, telomeres also contribute to the dynamic process of germ cell development (Reig-Viader et al. 2016; Keefe 2016). The following section will focus on recent insights on the role of the telomere structure in germ cell function. The chromatin dynamics in germ cells has been described elsewhere (Kota and Feil 2010; Gill et al. 2012).

In male germ cells, high telomerase activity is considered as a hallmark of undifferentiated spermatogonia (Pech et al. 2015). Then, telomerase activity progressively declines during gametogenesis and is absent in spermatozoa (Wright et al. 1996; Eisenhauer et al. 1997; Achi et al. 2000). Nevertheless, telomere length in spermatozoa is among the longest in mammalian cells. Importantly, it has been shown that sperm telomere length correlates with embryo quality and thus with good clinical outcome in in vitro fertilization procedures (Yang et al. 2015). Moreover, in late-generation telomerase-null mice, the germ cell compartment is strongly depleted, leading to almost empty seminiferous tubules (Lee et al. 1998). Thus, telomerase activity appears critical for male germ cell homeostasis, although it cannot be excluded that other lengthening mechanisms might take place during spermatogenesis (Tanemura et al. 2005). Conversely, and despite some discrepancies, telomeres in female germ cells do not elongate throughout oogenesis. Telomere length in oocytes is shorter than in somatic cells, most likely due to low or no telomerase activity and long exposure to reactive oxygen species before ovulation (Keefe et al. 2006; Liu et al. 2007).

Interestingly, in late-generation telomerase-null mice, meiosis is impaired due to altered synapsis of homologous chromosomes and decreased recombination (Liu et al. 2004b). Thus, proper telomere length homeostasis is essential for gametogenesis. During meiosis, telomeres tether chromosomes together to the nuclear envelope, leading to the formation of the “bouquet”, a characteristic structure of clustered telomeres (Scherthan 2007). This “bouquet” conformation promotes alignment and pairing of homologous chromosomes through cytoplasmic microtubule movements. Telomeres are attached to the nuclear membrane by their interaction with the linker of nucleoskeleton and cytoskeleton (LINC) complex that includes the highly conserved transmembrane proteins KASH5 and SUN1 (Shibuya and Watanabe 2014). The LINC complex connects telomeres to cytoplasmic motors (i.e., dynein and dynactin), allowing chromosome pairing (Ding et al. 2007). Telomere association with the LINC complex is mediated by the TERB1/2-MAJIN complex (Shibuya et al. 2015). TERB1 is a meiosis-specific protein with affinity for telomeric DNA (Daniel et al. 2014). In early prophase, a chimeric complex of Shelterin and TERB1/2-MAJIN is bound to telomeres. During meiosis progression, Shelterin is released from the complex, leaving only TERB1/2-MAJIN on the telomere. This meiosis-specific complex is essential for proper chromosome tethering and recombination, while protecting the chromosome ends. Therefore, and despite the fact that the Shelterin complex is considered as a hallmark of mammalian telomeres (Palm and de Lange 2008), in mature sperm cells, telomeres are specifically anchored to the nuclear periphery (Haaf and Ward 1995) through a sperm-specific protein complex that does not include TRF1 and TRF2, but contains a sperm-specific variant of histone H2B (Gineitis et al. 2000).

The higher order chromatin structure of sperm in mice specifies regulatory information to be used during embryonic development (Jung et al. 2017). However, it is not known whether there is structural information embedded in telomeric DNA that could be transmitted to the embryo. Indeed, only few data are available on the composition of telomeric chromatin in germ cells. TERRA is highly abundant in meiotic prophase I of both male and female human and mouse germ cells (Reig-Viader et al. 2014). TERRA precise role in this context is unclear, but it could be involved in telomerase regulation during gametogenesis or chromosomal movements, particularly homologous chromosome synapsis and pairing during meiosis. Indeed, in interphasic cancer cells, TERRA transcription correlates with higher telomere movements (Arora et al. 2012). Moreover, in somatic and ES cells, short telomeres or altered telomeric DNA structure can promote TERRA transcription (Schoeftner and Blasco 2008). Interestingly, in human somatic cells, H3K9me3 enrichment at telomeres might repress TERRA transcription, presumably through the action of SUV39H1 (Arnoult et al. 2012), while in human ES cells, reduction of TERRA expression impairs SUV39H1 recruitment and promotes telomere lengthening (Zeng et al. 2017). Thus, certain TERRA-dependent targeting of chromatin regulator might be cell specific. In mouse germ cells, loss of *Suv39h1* and *Suv39h2* results in impaired synapsis between homologous chromosomes, leading to spermatogenesis failure and infertility (Peters et al. 2001). However, this seems to be related to the loss of H3K9me3 at PCH and aberrant centromere clustering (Takada et al. 2011). Similarly, loss of *Setdb1*, another H3K9 methyltransferase, in the female germline leads to altered kinetochore-spindle interactions, bipolar spindle organization, and chromosome segregation defects (Kim et al. 2016; Eymery et al. 2016). However, the telomeric structure of *Setdb1* ^−/−^ germ cells was not specifically evaluated. Therefore, the precise molecular composition of telomeric chromatin in germ cells awaits future investigations.

#### Telomere dynamics during early development

Mammalian development starts by the fusion of two highly differentiated cells, the sperm and the oocyte. This event, commonly referred to as fertilization, marks the beginning of early embryonic development and is characterized by the formation of a totipotent 1-cell zygote. This unique cell will further divide and give rise to the whole embryo and extra-embryonic tissues, such as the placenta. As the embryo develops, the DNA methylation and histone PTM profiles at chromatin domains undergo extensive maturation, associated with changes in the expression of genes and repeated elements (Albert and Peters 2009). Proper and timed reorganization of the chromatin landscape in early embryos is required for their successful development (Beaujean 2014). Due to the limited number of available cells, the first studies were predominantly performed by microscopic analysis using indirect immunofluorescence or tagged proteins in combination with mouse genetic models (Santos et al. 2005; Burton and Torres-Padilla 2010). These studies revealed that broad genome domains show highly dynamic DNA methylation and histone PTM patterns during preimplantation development and that impairing the establishment of such patterns is deleterious for the embryo (Posfai et al. 2012). Due to their role in chromosomal stability and cellular homeostasis, it is important to understand telomere contribution and regulation during preimplantation embryo development.

First of all, the establishment of proper telomere length is of crucial importance for reproduction/fertility and embryo development. Knockout mouse models gave the first insights of telomere elongation role in mammalian development. Mice lacking the telomerase RNA component (*mTR* ^−/−^) do not show any obvious phenotype and can be maintained for at least six generations, suggesting that the overly long telomeres of laboratory mice could compensate for the telomeric sequence reduction. Nevertheless, telomeric DNA is gradually lost at each generation with increasing incidence of chromosomal abnormalities (Blasco et al. 1997), particularly in highly proliferative cells. Indeed, late-generation *mTR* ^−/−^ mice have hematopoietic defects, impaired mitogen-induced proliferation of primary splenocytes, and severe germ cell depletion (Lee et al. 1998). Male germ cells, which require a high level of telomerase activity for their efficient maintenance, are strongly depleted in these animals, resulting in decreased fertility (Lee et al. 1998; Pech et al. 2015). Although gametes from earlier generation *mTR* ^−/−^ mice (generation 2 or 3) can be used for in vivo or in vitro fertilization, they are less efficient than wild-type gametes and lead to aberrant embryos with pronucleus formation defects, cytofragmentation, and impaired preimplantation development (Liu et al. 2002). Of note, if short telomeres increase the risk of genomic instability, too long telomeric DNA also can compromise telomere stability and should similarly be avoided. In human and mouse ES cells, telomeric DNA trimming is performed by NBS1/XRCC3 (Rivera et al. 2016). Whether these complexes also regulate telomeric length during early embryonic development is currently not known.

**Fig. 2:**
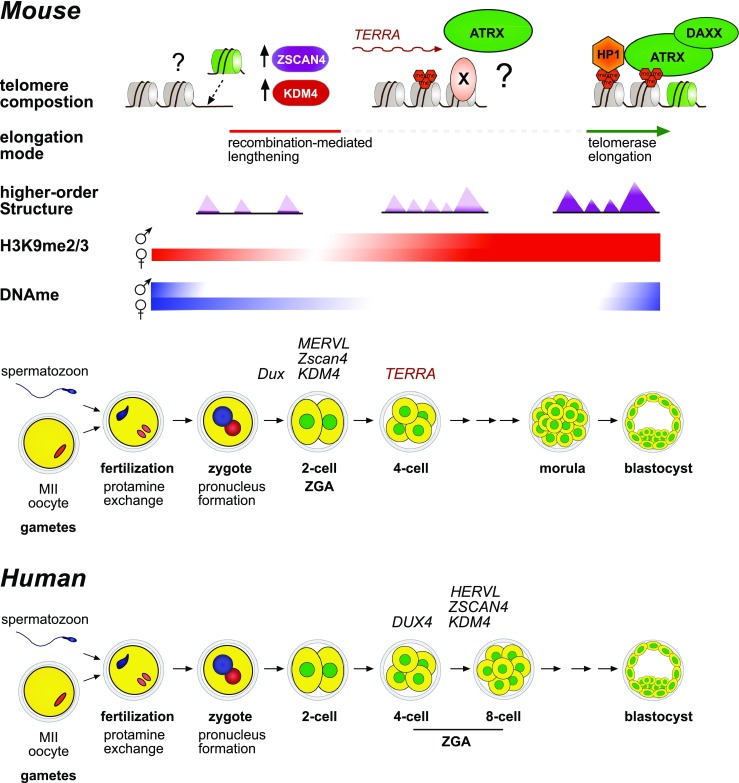
Telomere dynamics during mouse embryo preimplantation development. Extensive chromatin remodeling occurs at the onset of embryo development, with a histone-to-protamine replacement after fertilization, formation of the two parental pronuclei and major zygotic genome activation (ZGA) at the two-cell stage. In this environment, telomeres are elongated by a recombination-mediated mechanism that is telomerase-independent at the one- and two-cell stage. It is not known whether this mechanism is also used at later stages. Then, telomerase activity is readily detected starting from the morula-blastocyst transition. In mouse zygotes, ATRX labels PCH, and by the morula stage, ATRX is clearly targeted to telomeres. Telomere chromatin maturation might allow ATRX targeting by yet unknown mechanisms, such as recognition of histone PTM, a specific chromatin factor or binding to TERRA that is expressed at the 4-cell stage (TERRA dynamics at later stages are still unclear). DUX, a major driver of ZGA in both mouse and human zygotes, promotes expression of endogenous retroviruses (*MERVL*), H3K9 lysine demethylases from the *Kdm4* family and *Zscan4* that favors telomere elongation by recombination in mouse ES cells

Work from Liu and colleagues suggests that telomeres are elongated dramatically in the absence of any detectable telomerase activity after egg fertilization and activation (Liu et al. 2007). This surprising result warrants further investigations, as it implies the activation of an ALT-like mechanism at this developmental stage. During the first cell cycles, telomere length increases by several kilobases. This is incompatible with the sole activity of telomerase because it allows only the addition of a few hundred base pairs per division. Interestingly, early embryos show high T-SCE rate and co-localization of recombination proteins with TRF1 foci, a common phenotype of ALT cancer cells. In embryos, about half of the telomeres are localized close to PCH, while the other half is dispersed in the nucleoplasm, which is consistent with the acrocentric nature of mouse chromosomes (Aguirre-Lavin et al. 2012). These studies did not report any strong clustering of telomeres, which could be indicative of an ALT phenotype (Marzec et al. 2015). However, as embryonic chromatin is highly dynamic, these events might not be easily captured when using fixed material and might require live-imaging techniques. On the other hand, telomerase activity is clearly detected at the morula-blastocyst transition in mammals, when telomere length is stabilized (Wright et al. 1996; Schaetzlein et al. 2004). These intriguing observations suggest that the telomere maintenance mechanism switches from recombination-based “ALT-like” elongation after fertilization to telomerase activation prior to the blastocyst stage (Fig. 2). In human cancer cells, these two mechanisms are not always mutually exclusive. Perrem et al. (2001) and Cerone et al. (2001) describe co-occurrence of ALT with telomerase. In cancer cells, the ALT pathway generates heterogenous telomere length with very long and also very short telomeres. Such short telomeres could be generated by breakage in the telomere tract, which could either be extended by break-induced replication (BIR) or by the telomerase to ensure genome stability (Ford et al. 2001; Dilley et al. 2016). In contrast, it does not seem that very short telomeres are generated during early embryonic development, suggesting that the mechanism at play is not a typical ALT pathway. It could be that the transition between ALT-like and telomerase telomere lengthening might not be abrupt and that both mechanisms could co-exist during a short period in order to restore a homogeneous telomere length at the end of the preimplantation development. Moreover, it is not clear why and how telomeres are elongated by a telomerase-independent pathway in early embryos. Studies in cultured mammalian cells suggest that alteration of the telomere chromatin state could favor the ALT pathway (O’Sullivan and Almouzni 2014). Indeed, the chromatin landscape during early embryonic development is highly dynamic and more open than that of somatic cells (Wu et al. 2016). Single-nuclei Hi-C studies suggest that the chromatin of mouse preimplantation embryos exist in a “relaxed” state after fertilization, while higher-order structures are progressively re-established as embryonic development proceed (Du et al. 2017; Ke et al. 2017). Whether such “openness” of the genome at the early stages of development could favor the ALT pathway is unknown. Based on the present knowledge from studies of the ALT pathway in cancer cells and early embryonic development, several factors could contribute to telomere lengthening control in preimplantation embryos. Here, we will focus on the ATRX/DAXX complex and DUX/ZSCAN4 pathway (Fig. 2).

#### The ATRX/DAXX complex

As mentioned before, the ATRX/DAXX complex is a key repressor of the ALT pathway. Mouse *Atrx* or *Daxx* genetic ablation is embryonic lethal at E9.5 (Michaelson et al. 1999; Garrick et al. 2006). *Daxx* ^*−/−*^ embryos are growth-retarded and show massive apoptosis, while embryos in which *Atrx* is inactivated at the 8–16-cell stage grow and implant normally, but die later because of defects in extraembryonic trophectoderm formation. The presence of defects in telomere maintenance in these embryos was not determined. *Atrx* and *Daxx* mRNA are maternally provided in the embryo (RNA sequencing data from Park et al. 2013). Interestingly, ATRX and DAXX undergo a spatiotemporal re-localization during preimplantation embryo development. After fertilization, ATRX is localized at maternal condensed chromosomes, then it labels predominantly PCH domains (which assembles around prenucleolar bodies) of maternal origin where it represses transcription of the underlying major satellite repeats (De La Fuente et al. 2015). Conversely, DAXX is concentrated on paternal PCH, defining an interesting epigenetic asymmetry that contrasts with the classic view of ATRX and DAXX co-localization at these regions, as observed in oocytes and most somatic cells. Interestingly DAXX targeting at IAP retrotransposons in ES cells does not rely on ATRX (Hoelper et al. 2017). Thus, ATRX and DAXX could operate in different complexes during development and assemble on chromatin with different kinetics. Strikingly, by the morula stage, ATRX and DAXX are mostly co-localized at telomeres (He et al. 2015). Therefore, ATRX/DAXX absence from telomeres in the early stages of development is compatible with an ALT-like mechanism and suggests that telomeric chromatin undergoes changes during development, thus allowing the specific loading of ATRX and DAXX at the end of preimplantation. The reasons for ATRX and DAXX absence at telomeres in the early stages of embryo development are unclear. Specific requirements for ATRX targeting (i.e., H3K9me3 or HP1 proteins) could be missing on telomeric chromatin just after fertilization and be re-established only later during development. The paternal genome undergoes massive protamine-to-histone exchange right after fertilization and this could impair ATRX loading. At the one- and two-cell stage, H3K9me2/3 is low on the paternal pronucleus and global levels on both pronuclei start to increase at the four-cell stage onward (Liu et al. 2004a; Yeo et al. 2005). Such dynamics in the H3K9 methylation could thus favor ATRX loading at telomeres only latter during development after histone methylation levels are restored. Also, it would be interesting to test whether the kinetics of ATRX and DAXX loading at telomeres differs according to the parental origin (telomere transgenerational inheritance and parental origin are discussed below).

Similarly, it is not known how ATRX and DAXX are loaded at telomeres later in development. It was suggested that in mouse ES cells, DNA hypomethylation promotes ATRX/DAXX loading at tandem repeats (e.g., IAP or telomeres). This could lead to SUV39H1 recruitment to mediate H3K9me3 and heterochromatinization (He et al. 2015). Such model is appealing because the genome undergoes global hypomethylation during preimplantation and this could favor ATRX binding (Messerschmidt et al. 2014). However, telomeres and IAP are not regulated by DNA methylation in ES cells, and IAP retains high levels of DNA methylation throughout preimplantation (Maksakova et al. 2013; Arand et al. 2015). Furthermore, subtelomeric DNA methylation is not altered in induced pluripotent stem (iPS) cells generated from mouse embryo fibroblasts, suggesting that, at least during in vitro reprogramming, DNA methylation is maintained at these loci (Marion et al. 2009). Whether this also applies to preimplantation embryos is not clear and will require further investigations.

During early embryo development, several transposable elements and tandem repeats undergo massive transcription (Peaston et al. 2004; Probst et al. 2010; Fadloun et al. 2013). As exemplified with PCH, transcription of major satellites is required for efficient maturation of these loci and embryo development progression (Casanova et al. 2013). TERRA signals are detected by RNA FISH by the 4-cell stage (Probst et al. 2010). We could hypothesize that TERRA transcription in the embryo represents a starting signal for recruiting ATRX and/or other chromatin factors to telomeres and establishing a canonical H3K9me3/HP1 heterochromatic state (Nguyen et al. 2017). Thus, deciphering the regulation of ATRX/DAXX recruitment at telomeres during natural embryo development could help better understanding heterochromatin establishment at telomeric DNA.

#### The DUX/ZSCAN4 pathway

After fertilization, maternally inherited transcripts control development before their rapid clearance. The embryonic genome is then activated (zygotic genome activation (ZGA)), marking the maternal-to-zygotic transition (Svoboda et al. 2015). In mice, a minor ZGA occurs at the one-cell stage followed by a major ZGA at the two-cell stage (four- to eight-cell stage in humans). Zinc finger and SCAN domain-containing 4 (*Zscan4*) is among the genes that are specifically expressed during ZGA (Falco et al. 2007) (Fig. 2, bottom panel). In mice, the *Zscan4* cluster includes six genes and three pseudogenes, while humans only have one copy of *ZSCAN4*. In preimplantation embryos, *Zscan4* expression peaks at the two-cell stage in mice and at the four- to eight-cell stage in humans (Vassena et al. 2011; Ishiguro et al. 2017). Interestingly, a subpopulation of proliferating mouse ES cells (about 1–5% of all cells) expresses transiently transcripts that are detected in two-cell embryos, such as the MERVL endogenous retrovirus and the *Zscan4* cluster. Accordingly, these cells are referred to as “2C-like” cells (Macfarlan et al. 2012). ES cells that express *Zscan4* (“Zscan4-associated event” or “Z4 event”) are characterized by rapid and telomerase-independent telomere lengthening. Telomeres are bound by recombination proteins and undergo T-SCE (Zalzman et al. 2010). In ALT cancer cells these features are associated with genomic instability. Conversely, *Zscan4* expression in ES cells is associated with improved karyotypes and developmental potency and is required for long-term culture of ES cells (Amano et al. 2013). Similarly, *Zscan4* improves iPS cell reprogramming efficiency, when expressed for only a few days (Hirata et al. 2012; Jiang et al. 2013). However, uncontrolled *Zscan4* expression leads to over-recombination, telomere length heterogeneity, and chromosome fusions (Dan et al. 2014). In preimplantation mouse embryos, *Zscan4* genetic ablation or sustained expression impairs development progression and blastocyst implantation (Falco et al. 2007). Thus, fine-tuned *Zscan4* expression is required for proper genome stability in ES cells and early embryo development.

Interestingly, the chromatin state of ES cells that express *Zscan4* is characterized by global DNA hypomethylation, histone hyperacetylation, and transcription of heterochromatin domains (pericentromeres, telomeres, and retrotransposons) (Akiyama et al. 2015; Eckersley-Maslin et al. 2016). Hypomethylation of subtelomeric DNA is associated with recombination-mediated telomere lengthening, while hypermethylation, as observed in *Tet* ^−/−^ ES cells, is associated with reduced recombination and telomere shortening (Gonzalo et al. 2006; Yang et al. 2016). *Tet1* knockdown in epiblast-like cells (EpiLC) results in *Zscan4* decrease and telomere shortening, while *Tet* TKO in ES cells cultured in 2i conditions (“ground state”) induces increased *Zscan4* expression and telomere elongation (Lu et al. 2014; Khoueiry et al. 2017). Thus, Tet enzymes may regulate *Zscan4* levels with different outcomes depending on the cell type or pluripotency status. Accordingly, the hypomethylated state of *Zscan4*-expressing cells should favor recombination-mediated telomere elongation. The expression and localization of activating and repressive chromatin regulators in ZSCAN4-positive cells suggest that ZSCAN4 and, most likely, other factors promote first an open and then repressed chromatin state, presumably to allow the transient nature of the “2c-like” state in ES cells (Akiyama et al. 2015). Indeed, a prolonged “2c-like” state in ES cells might lead to the irreversible erasure of specific chromatin marks, such as parental imprints (Eckersley-Maslin et al. 2016).

How are *Zscan4* genes induced during ZGA? In ES cells, the transcription factor TBX3 induces *Zscan4* expression through modulation of DNMT3b and TET2 levels, thereby reducing DNA methylation at the *Zscan4* locus (Dan et al. 2013). However, single-cell transcriptomic data in preimplantation mouse embryos suggest that *Tbx3* expression is restricted to the inner cell mass (Nestorov et al. 2015). Therefore, a *Tbx3* role in *Zscan4* expression at the two-cell stage seems unlikely. Several studies identified human *DUX4* and mouse *Dux* genes as master regulators of many genes and retrotransposons, including *Zscan4*, during ZGA (De Iaco et al. 2017; Hendrickson et al. 2017; Whiddon et al. 2017). What drives the expression of *Dux/DUX4* is still unclear. Its expression appears to be associated with chromatin relaxation or destabilization, as observed in mouse ES cells in which the chromatin assembly factor (CAF-1) *Chaf1a* is downregulated and in myoblasts from patients with FSHD (Geng et al. 2012; Ishiuchi et al. 2015). The early embryo genome is characterized by an open chromatin structure and dynamic histone mobility (Bošković et al. 2014; Wu et al. 2016). This environment could favor *Dux/DUX4* upregulation and consequently *Zscan4* gene transcription to promote a rapid, but transient telomere lengthening and karyotype correction. Maternally and/or zygotically expressed factors that repress the 2c-like state in ES cells (such as SETDB1, HP1, and TRIM28) could participate in *Dux* and *Zscan4* repression and allow the establishment of a canonical heterochromatin state at later developmental stages (Macfarlan et al. 2012; Maksakova et al. 2013; De Iaco et al. 2017).

#### Chromatin marks at telomeres and genome reprogramming

After fertilization, the embryo genome undergoes a rapid reorganization or “reprogramming” that is essential for proper ZGA, totipotency acquisition, and development. Nuclear reprogramming can by studied by somatic cell nuclear transfer (SCNT) in which a donor nucleus (usually from a somatic cell) is injected into an enucleated oocyte. The oocyte will reprogram the donor nucleus in a similar way as the reprogramming of the maternal and paternal genomes after natural fertilization (Sepulveda-Rincon et al. 2016). Another method consists in expressing keys transcription factors (*Oct4*, *Sox2*, *Klf4*, and *Myc*) in somatic cells to induce pluripotency (Takahashi and Yamanaka 2016). In this way, iPS cells have been successfully generated in different mammalian species. However, experimental reprogramming is not equivalent to what observed following natural fertilization, suggesting that somatic cells contain specific factors or chromatin determinants that are refractory to artificial reprogramming. Telomere rejuvenation is a feature of successful reprogramming and its efficiency can be enhanced by specific alterations of the chromatin state (Liu 2017). Strikingly, H3K9 methylation appears to be a roadblock to efficient reprogramming in both SCNT and iPS cells (Chen et al. 2013; Matoba et al. 2014; Chung et al. 2015). During iPS cell induction, telomeres adopt a chromatin structure similar to that of ES cells with reduced H3K9me3 (Marion et al. 2009). Reduction of H3K9me3 levels by ectopic expression of the H3K9 demethylase KDM4B in SCNT-derived embryos helps to restore transcriptional reprogramming and induces *Zscan4* expression (Matoba et al. 2014). Moreover, *Kdm4b* or *Zscan4* expression enhances telomere elongation and the successful formation of iPS cells (Jiang et al. 2013; Sridharan et al. 2013). In pluripotent cells, SUV39H1/2 and SETDB1 could control H3K9me3 levels at telomeres (García-Cao et al. 2004; Udugama et al. 2015). Both maternally inherited *Suv39h2* and *Setdb1* transcripts are found in the zygote (Park et al. 2013). However, the absence of ATRX binding to telomeres, which relies predominantly on H3K9me3 and HP1, suggests that H3K9me3 methyltransferases are not targeted to these regions in the zygote. Genome-wide studies of the chromatin landscape in early embryos will surely provide a better understanding of the dynamic organization of telomeres during preimplantation development.

#### Inheritance and parental origin

The discovery that after the histone to protamine exchange, the fraction of histones retained in mature sperm carries PTM led to the idea that male germ cells could transmit epigenetic information to the progeny (Gill et al. 2012). Moreover, the retained histones are not randomly positioned in the genome. Indeed, ChIP-sequencing analysis of nucleosomes and histone PTM in human and mouse mature sperm cells led to the identification of a specific chromatin structure at developmentally regulated genes (Hammoud et al. 2009; Brykczynska et al. 2010; Erkek et al. 2013). Removal of histone marks from sperm impairs the expression of embryonic genes in a paternal-specific manner and leads to impaired embryo development (Teperek et al. 2016). Chromatin marks and more generally higher-order chromatin structures in sperm cells (i.e., 3D organization, enhancers) are supposed to influence genome organization and gene expression in the embryo (Jung et al. 2017). Several studies reported that telomeres in human mature sperm cells contain nucleosomes (Zalenskaya et al. 2000), and that nucleosomes can be found in gene-poor regions and at several repeated elements in sperm (Carone et al. 2014; Samans et al. 2014). However, bioinformatics analysis of repeated elements is to be considered very carefully because the obvious repetitive nature of these elements requires proper data treatment to prevent misleading conclusions (Royo et al. 2016). Moreover, chromatin higher-order structure of both parental genomes is drastically remodeled from fertilization until blastocyst stage (Du et al. 2017). Therefore, it is not clear whether in gametes, telomeres have a specific chromatin structure that could be inherited and with a role in early development.

The parental origin influence, if any, on telomeric chromatin regulation is also quite enigmatic. A parental-specific chromatin structure is visible at PCH domains in the zygote. H3K9me3 and HP1 are absent from paternal PCH, while they are highly enriched at maternal PCH. Conversely, Polycomb complexes bind to these domains and repress expression of major satellites from the paternal pronucleus (Puschendorf et al. 2008). To analyze the influence of the parental origin on telomere regulation, Liu and colleagues analyzed telomere lengthening in mouse parthenotes (embryos with a genome of only maternal origin). Telomere elongation was comparable in parthenotes and in in vitro fertilized zygotes, suggesting that the factors controlling telomere lengthening in embryos are provided by the oocyte (Liu et al. 2007). Interestingly, ATAC-seq and DNAse-seq genomic analyses, which provide allelic information in early embryos, suggest that chromatin accessibility is comparable in the two parental genomes with the exception of imprinted genes (Wu et al. 2016; Lu et al. 2016). Therefore, paternal chromatin accessibility is reprogrammed shortly after fertilization to similar levels as for maternal chromatin. This suggests that the parental-specific differences in chromatin states of telomeres, if they exist, do not exert a strong influence on their regulation at the onset of development.

## Conclusions

Telomeres are essential structure to ensure genome stability, tissue homeostasis, and embryo development. Telomeric chromatin plasticity is a striking feature that allows the dynamic regulation of chromosome ends following developmental cues, cell cycle, or metabolism. These plasticity features are crucial during natural development or experimental reprogramming of somatic nuclei. Thus, understanding the mechanisms that operate at telomeric DNA in these conditions will greatly help the development and optimization of assisted reproductive technologies or therapeutic reprogramming techniques (Sepulveda-Rincon et al. 2016). Furthermore, chromatin modifications at telomeric repeats can alter the telomere lengthening mode, switching to the ALT pathway (O’Sullivan and Almouzni 2014). Although telomerase is the most common mechanism of telomere maintenance in cancer cells, treatment with telomerase inhibitors can lead to the appearance of ALT (Hu et al. 2012). Thus, understating how the chromatin state can promote one or the other telomere maintenance mode could lead to the development of therapeutic strategies to enhance anti-telomerase treatments. Interestingly, ALT or ALT-like lengthening also occurs in physiological conditions (Liu et al. 2007; Neumann et al. 2013). Deciphering the role of such mechanisms during evolution and comparing the mechanisms used in other organisms should provide clues on the advantages of using a specific telomere-lengthening mode during the development and lifespan of an organism.
